# Comparisons of Physicians’, Nurses’, and Social Welfare Professionals’ Experiences With Participation in Information System Development: Cross-Sectional Survey Study

**DOI:** 10.2196/51495

**Published:** 2025-01-22

**Authors:** Susanna Martikainen, Johanna Viitanen, Samuel Salovaara, Ulla-Mari Kinnunen, Tinja Lääveri

**Affiliations:** 1 Department of Health and Social Management, Faculty of Social Sciences and Business Studies, University of Eastern Finland, Kuopio, Finland; 2 Department of Computer Science, School of Science, Aalto University, Espoo, Finland; 3 Department of Social Work, Faculty of Social Sciences, University of Lapland, Rovaniemi, Finland; 4 Infectious Diseases, Inflammation Center, University of Helsinki and Helsinki University Hospital, Helsinki, Finland

**Keywords:** participation, development, usability, user experience, physician, nurse, social worker, information system, national survey, system development, users, user feedback, cross-sectional survey, Finland, Finnish

## Abstract

**Background:**

The integration of health care and social welfare services together with the consolidation of health care information systems (HISs) and client information systems (CISs) has become a timely topic. Despite this development, there is a scarcity of systematic research on physicians’, registered nurses’ (RNs) and social welfare professionals’ (SWPs) experiences of participating in the development of HISs and CISs.

**Objective:**

This study aimed to examine how physicians, RNs and SWPs experience collaboration with HIS or CIS vendors, and what kinds of end users have participated in HIS or CIS development.

**Methods:**

National cross-sectional usability surveys were conducted in Finland among RNs and SWPs in 2020 and physicians in 2021. Questions concerning participation experiences were analyzed by professional group, working sector, managerial position, and age.

**Results:**

In total, 4683 physicians, 3610 RNs, and 990 SWPs responded to the surveys. In all 3 professional groups, those working in nonmanagerial positions and the youngest respondents participated least in HIS or CIS development, and 76% (n=3528) of physicians, 78% (n=2814) of RNs and 67% (n=664) of SWPs had not participated at all. When comparing the groups, physicians were least aware of feedback processes and least satisfied with vendors’ interest in end-user feedback and the manner and speed of HIS development. Those who had dedicated working time for HIS or CIS development were less critical of vendors’ interest and responsiveness to development ideas than those who had not participated at all. In all 3 professional groups, the youngest were most dissatisfied with HIS and CIS vendor collaboration.

**Conclusions:**

Experiences of participation in HIS and CIS development were relatively negative across all 3 professional groups, with physicians being the most critical. Dialogue and collaboration between developers and end users—also the youngest ones and frontline workers—need improvement; simply increasing allotted working time is unlikely to produce more positive participation experiences.

## Introduction

### Background

The increasing collaboration between health care and social care and the consequent need for integrated information systems (ISs) warrants studies on the end-user participation viewpoints of all major user groups. Physicians [1-5], nurses [1,2], and social welfare professionals (SWPs) [6] are not satisfied with the usability or daily work support of health care information systems (HISs) and client information systems (CISs) [7]. Furthermore, these professionals experience collaboration with HIS and CIS vendors and developers to be unsatisfactory. However, the majority would be willing to participate in information system development, but suitable means are lacking [1,2,6,8-11].

End users’ participation in IS development is considered to lead to a better user experience and increased user acceptance [12,13]. However, the characteristic inherent complexity of HISs and CISs complicates the implementation of many user participation methods [14,15]. Although end-user participation is regarded as essential in HIS and CIS development [16], without careful management throughout the software development process, participation alone does not guarantee system success [17-19]. End-user participation may even cause more problems than benefits, particularly if the ISs are expected to solve organizational problems [20]. Users may experience that their participation does not affect IS functionalities in a desired manner [1,4,6,16]. In addition, emphasizing administrative information needs and wishes instead of the needs of frontline professionals can complicate work processes by increasing the requirements for data entry and thus impair the workflows [21]. IS users in leadership positions have different needs for ISs than frontline professionals [22].

Comprehensive HISs and CISs are complex ISs used by dozens of user roles in a wide variety of use contexts [23,24]. Many countries, including Finland, are integrating health and social welfare services and consequently ISs [25-27]. One of the rationales behind this development is that those with high numbers of visits to health care often need social welfare services and vice versa [28]. From the point of view of patients and clients, treatment and service packages often include both health care and social services [29-33], which emphasizes the need for fluent information exchange between professional groups to guarantee high-quality and safe care. Consequently, end users from the major professional groups are needed in the IS development processes.

### Context of the Study: Health Care, Social Welfare and HISs and CISs in Finland

Until 2023, municipalities (n=309 in 2022) were responsible for organizing social welfare services and primary health care (health centers) in Finland. A total of 20 hospital districts, jointly owned by the municipalities of the region, organized specialized medical care; 5 university hospitals provided tertiary care. Although one-third of outpatient visits to physicians are to private providers (eg occupational health care), the variety of services provided by the private sector is narrow; for example, there are no private intensive care units (ICUs) or labor and delivery units [34]. In social welfare, municipalities or federations of municipalities often purchase some services from private service providers and non-governmental organizations (n=3971 in 2017) [27]. In 2018, there were 19,627 working-age physicians, 70,198 RNs, and 34,523 SWPs in Finland [35-37].

In Finland, the first HISs and CISs were implemented in the 1970s [27]. While only every tenth US hospital used electronic health records (EHRs) as late as in 2010 [38], in Finnish public health care, HIS coverage had already reached 100% by 2007, and by 2014 CISs covered almost all public social services [39,40]. By contrast, in 2020, a quarter of nonpublic social welfare organizations still operated on paper [27,41].

All public hospitals and health centers had joined the Kanta services (national patient data repository and electronic prescription system) by 2015 [42,43], but implementation of the national data repository for social welfare services only began in 2020 [44]. This has required considerable resources from both health care and social welfare organizations and IS vendors over the years [45].

During 2020‐21, in public health care, 2 leading specialized care and 2 leading primary care EHR brands were in wide use. In addition, 4 EHR brands covered both primary and specialized care, of which 1 also covered tertiary care (including functionalities for eg, operating rooms, ICUs, radiology, and emergency departments) and 6 out of the 7 nationally defined social welfare service lines. In addition, 1 EHR brand covered most private sector health care. In public social welfare, 2 CIS brands were in use in most municipalities [46]. In addition, EHR brands were also used in social welfare [41]. In 2018‐21, a new IS was deployed in Southern Finland with 47,000 end users.

### Research Questions

In this study, we examined the experiences of physicians, registered nurses (RNs) and SWPs of participating in HIS or CIS development. The data were gathered in 3 large Finnish national surveys in 2020 and 2021. The research questions were as follows:

1. What experiences do physicians, RNs and SWPs have of collaboration with HIS and CIS vendors?
2. Do participation experiences vary by managerial position, employment sector, or age?
3. What types of physicians, RNs and SWPs have participated in HIS and CIS development?

## Methods

### Survey

This study was part of large national cross-sectional HIS and CIS usability surveys conducted among SWPs and RNs in 2020, and physicians in 2021 [47]. The survey questionnaires and data are available online [47]. The surveys were based on the validated National Usability-Focused HIS Scale [48] and included a section on end users’ experiences of participation in HIS or CIS development (Table 1). The statements were originally created and piloted for the national physician survey in 2010 [4,5], and the same statements have been used in later surveys for physicians [1], for RNs [8,49], and SWPs [6]. The survey method and the questionnaire have been described in detail previously [4,5,48].

**Table 1.** Questionnaire statements.

Questions and statement/option designations		Statements and options
Question (1) What has been your experience of providing feedback on the information systems you use and their development? please assess the following statements based on your experience. Response options: Fully agree / Somewhat agree / Neither agree nor disagree / Somewhat disagree / Fully disagree		
	Statement A	I know how and to whom I can send feedback about the system if I wish to do so.
	Statement B	The system vendor is interested in feedback about the system provided by the end users.
	Statement C	The system vendor implements corrections and change requests according to the suggestions of the end users.
	Statement D	Corrections and change requests are implemented within a reasonable time frame.
Question (2) Have you participated in information systems development work?		
	Option A	Yes, some of my working time has been allocated for such development work
	Option B	Yes, in addition to my work
	Option C	No

The link to the survey questionnaire was sent to the members of the Finnish Medical Association (FMA) (>90%) (email address available, n=19,142), RN members of the Finnish Nursing Association, the National Association of Health and Welfare Professionals, and the National Professional Association (n=58,276), and SWPs with at least a Bachelor’s degree who were members of the following trade unions: the Union of Professional Social Workers, the Trade Union for the Public and Welfare Sectors, or the Social Science Professionals union (n=12,471) [9,50,51] .

**Figure 1.**
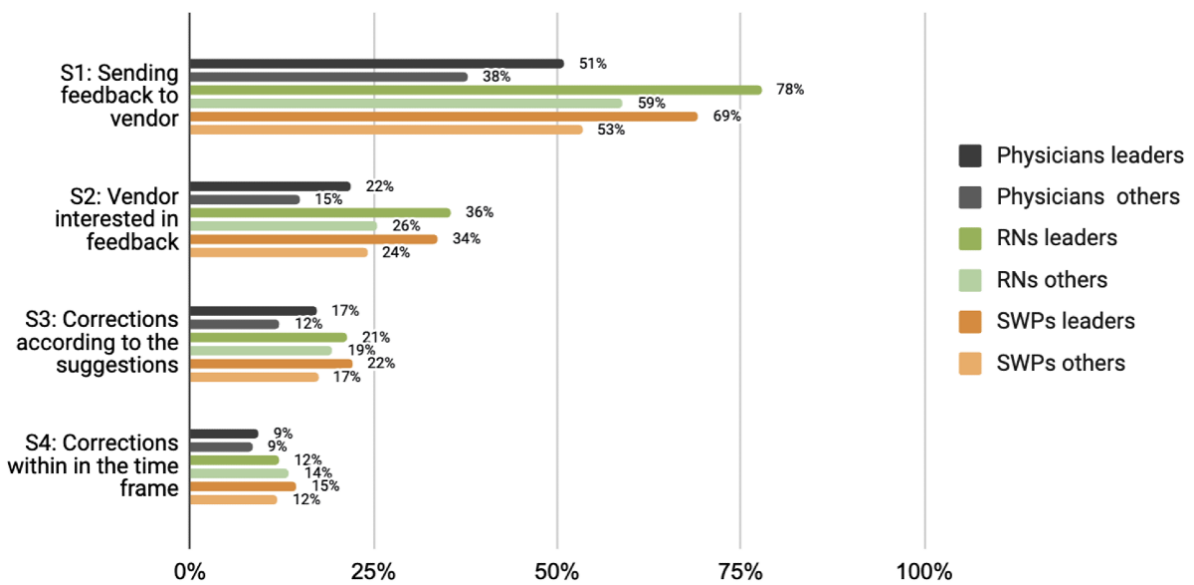
Participation experiences of Finnish physicians, RNs and SWPs by leadership position (leaders versus others). RN: registered nurse; SWP: social welfare professional.

The section addressing end-user participation experiences in HIS or CIS development (Table 1) was identical for the physicians and the SWPs with 5-point Likert scale response statements (fully agree; somewhat agree; neither agree nor disagree; somewhat disagree; or fully disagree). However, for the 2020 RNs’ survey, a sixth option “Prefer not to respond / Don’t know” was added. Furthermore, unlike in the physicians’ and SWPs’ surveys, it was not possible for RNs to not respond at all. Up to 25% of RNs chose this sixth option. To make the surveys more comparable, we formed a sixth category also for physicians and SWPs of those who had not responded to the statements. In addition, for the descriptive statistics in Figures1^4^ and Table S1 in Multimedia Appendix 1, we combined the responses “Fully agree” and “Somewhat agree” to form a new category “Agree” and included those from the sixth category in the denominator.

The statement on having allocated time to participate in IS development was identical in all 3 surveys, and the background questions included in this study were all optional in all 3 surveys, so nonrespondents were not included in these numbers.

**Figure 2.**
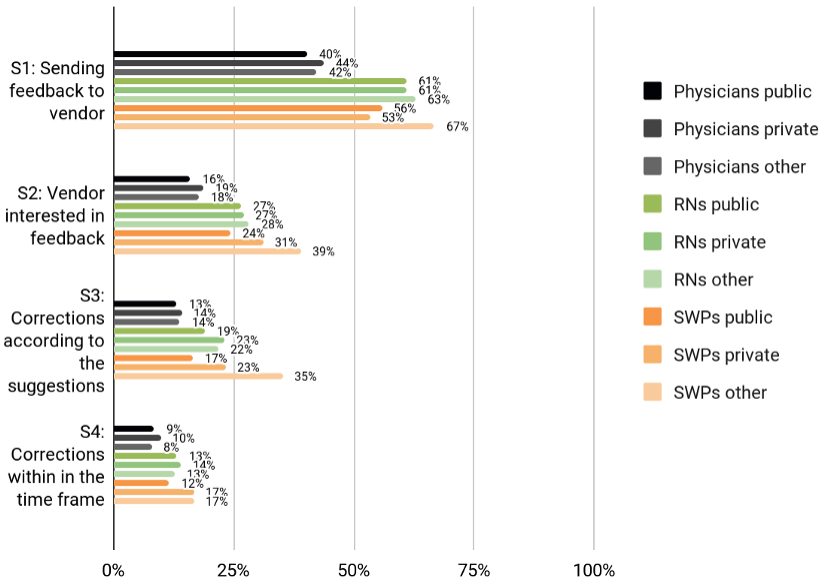
Participation experiences of Finnish physicians, RNs and SWPs by working sector. RN: registered nurse; SWP: social welfare professional.

**Figure 3.**
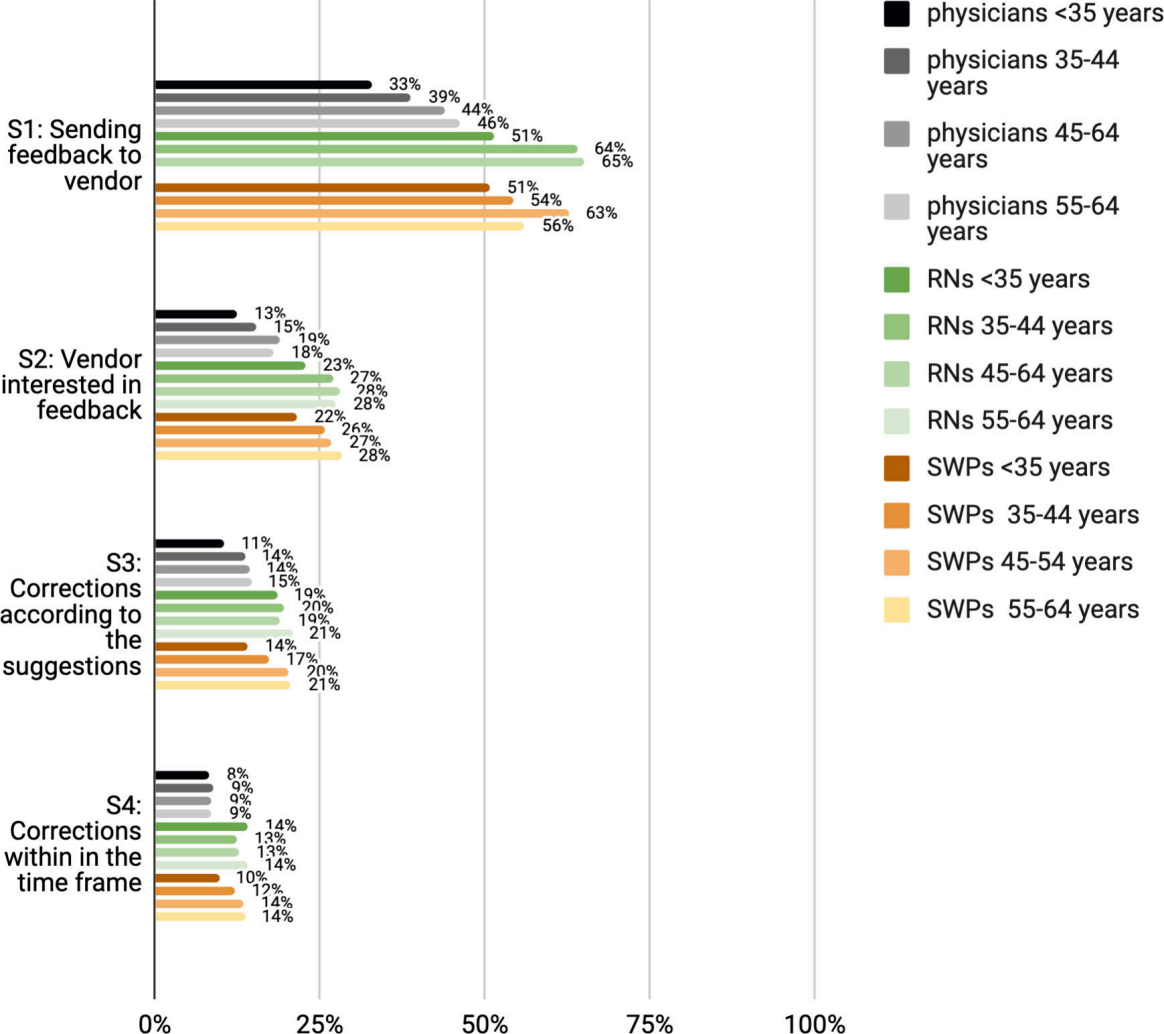
Participation experiences of Finnish physicians, RNs and SWPs by age group. RN: registered nurse; SWP: social welfare professional.

**Figure 4.**
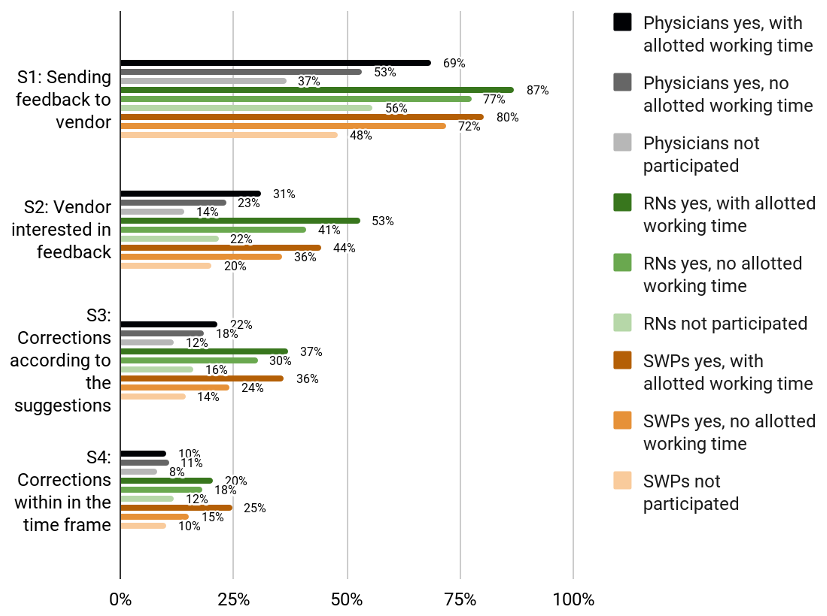
Participation experiences of Finnish physicians, RNs and SWPs by working time allotted for participation. RN: registered nurse; SWP: social welfare professional.

### Statistical Analyses

Statistical analyses were carried out with SPSS 28 (IBM Corp). The *χ*² test or Fisher exact test was used as appropriate. Statistical significance was determined as *P*<.05.

### Ethical Considerations

According to the national ethical instructions for research, the studies did not require ethical approval (Finnish Advisory Board on Research Integrity 2023) [52].

The autonomy of research subjects was respected, there was informed consent, no harm was possible to the participants and confidentiality of the subjects, and research data were protected. The researchers were not able to identify individual respondents. However, as the data for the RN and SWP studies were collected by a national authority (Finnish Insititute for Health and Welfare), the ethical approval (THL482/6.02.01/2020) was provided by its institutional review board.

## Results

### Respondent Characteristics

The demographics of the respondents to all 3 surveys are provided in Table 2. In 2021, 4683/19,142 physicians (24.5% of email invitation recipients) participated in the survey, and in 2020, 3610/58,276 RNs (6.2% of email invitation recipients) and 990/12,471 SWPs (7.9% of the theoretical target group) participated in the survey [35-37].

**Table 2.** Respondent characteristics.^a^

	Physicians (n=4683), n (%)	Registered nurses (n=3610), n (%)	Social welfare professionals (n=990), n (%)
Working sector			
Public sector	3654 (78)	3076 (85.2)	846 (85.5)
Private	775 (16.5)	456 (12.6)	90 (9.1)
Other	253 (5.4)	78 (2.2)	54 (5.5)
Age group (years)			
<35	949 (20.3)	739 (20.5)	185 (18.7)
35‐44	1215 (25.9)	833 (23.1)	346 (34.9)
45‐54	1161 (24.8)	1108 (30.7)	260 (26.3)
55‐64	1315 (28.1)	921 (25.5)	198 (20)
Leadership position			
Works in a leading or managerial position	1139 (24.3)	406 (11.2)	172 (17.4)
Works in other positions	3543 (75.7)	3204 (88.8)	818 (82.6)

a The email invitation was received by 19,142 physicians, 58,276 registered nurses, and 12,471 social welfare professionals. Of these, 4683 (24.5%) physicians, 3,610 (6.2%) registered nurses, and 990 (7.9%) social welfare professionals responded to the survey.

### Participation Experiences of the 3 Professional Groups

**Figure 5.**
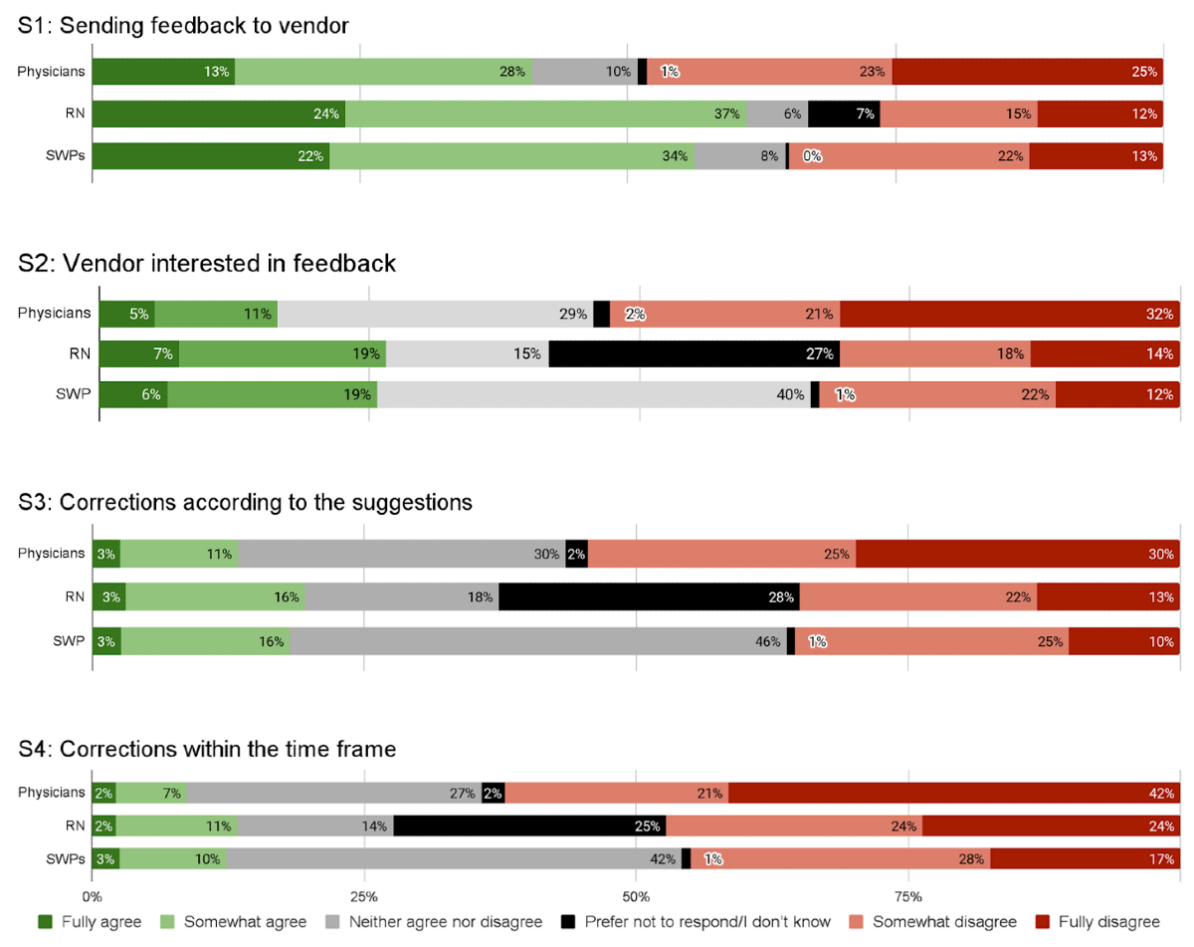
Experiences of physicians, RNs and SWPs of collaboration with health care information system and client information system developers. RN: registered nurse; SWP: social welfare professional.

**Figure 6.**
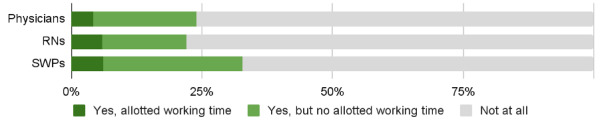
Participation in information system development work by professional group. RN: registered nurse; SWP: social welfare professional.

Physicians appeared to be least knowledgeable of how and where to send feedback, with only 41% (1920/4683) agreeing with statement 1, as compared to 61% (2204/3610) of RNs and 56% (556/990) of SWPs (Figure 5). In terms of vendors’ interest in end-user feedback and the manner and speed of IS development, physicians were also least satisfied, with 16% (776/4683), 14% (672/4683), and 9% (407/4683) of them, respectively, agreeing with statements 2, 3, and 4, as compared to 26% (960/3610), 19% (707/3610), and 13% (481/3610), respectively, of RNs and 25% (225/990), 19% (180/990), and 13% (122/990), respectively, of SWPs (Figure 5). In total, 76% (3528/4683) of physicians, 78% (2814/3610) of RNs and 67% (664/990) of SWPs had not participated at all in HIS or CIS development (Figure 6).

### Factors Associated With Participation Experiences

Leaders, particularly among RNs, were more aware of how and where to send system feedback (statement 1) than those working in nonmanagerial positions (Figures1^4^). Leaders in all 3 professional groups were more satisfied with the system vendor collaboration (statements 2‐4) than the others. The working sector did not impact end users’ experiences. In all professional groups, the youngest appeared least aware of how and where to send feedback and least satisfied with the collaboration. Those who had participated in HIS or CIS development considered IS vendors more interested in feedback and were more satisfied with the manner and speed of system improvements and corrections.

### Factors Associated With Having Participated in HIS or CIS Development

In all 3 professional groups, leaders had participated more in IS development than their colleagues in nonleadership positions (Figure 6).

Among physicians and RNs, but not SWPs, those working in the private sector had participated less than their public sector colleagues (Table 3). In all 3 professional groups, the youngest had participated the least (Table 3).

**Table 3.** Participation in development by allocated working time.

Participated in development		Yes, allotted working time, n (%)^a^	Yes, but no allotted working time, n (%)^a^	Not at all, n (%)^a^
Leadership position				
Physicians				
Leaders		85 (7.5)	403 (35.7)	640 (56.7)
Others		115 (3.3)	514 (14.6)	2887 (82.1)
RNs^b^				
Leaders		54 (13.3)	131 (32.3)	221 (54.4)
Others		162 (5.1)	449 (14)	2593 (80.9)
SWPs^c^				
Leaders		11 (6.4)	83 (48.3)	78 (45.3)
Others		50 (6.1)	180 (22.1)	586 (71.8)
Working sector				
Physicians				
Public		161 (4.4)	764 (21)	2705 (74.5)
Private		26 (3.4)	104 (13.5)	639 (83.1)
Other		13 (5.3)	49 (20)	183 (74.7)
RNs				
Public		188 (6.1)	500 (16.3)	2388 (77.6)
Private		23 (5)	65 (14.3)	368 (80.7)
Other		5 (6.4)	15 (19.2)	58 (74.4)
SWPs				
Public		53 (6.3)	218 (25.8)	573 (67.9)
Private		6 (6.7)	24 (26.7)	60 (66.7)
Other		2 (3.7)	21 (38.9)	31 (57.4)
Age group (years)				
Physicians				
Age group<35		20 (2.1)	93 (9.9)	827 (88)
Age group 35‐44		59 (4.9)	227 (18.8)	920 (76.3)
Age group 45‐54		68 (5.9)	299 (25.9)	787 (68.2)
Age group 55‐64		52 (4)	289 (22.2)	962 (73.8)
RNs				
Age group<35		34 (4.6)	89 (12)	616 (83.4)
Age group 35‐44		53 (6.4)	134 (16.1)	646 (77.6)
Age group 45‐54		83 (7.5)	186 (16.8)	839 (75.7)
Age group 55‐64		45 (4.9)	166 (18)	710 (77.1)
SWPs				
Age group<35		13 (7.1)	32 (17.5)	138 (75.4)
Age group 35‐44		23 (6.6)	97 (28)	226 (65.3)
Age group 45‐54		13 (5)	84 (32.3)	163 (62.7)
Age group 55‐64		12 (6.1)	50 (25.3)	136 (68.7)

a Denominators for calculating percentages are the sum of n values for each row

b RN: registered nurse

c SWP: social welfare professional

## Discussion

### Overview

To our knowledge, this is the first study to assess how the major professional groups in the health care and social welfare sector, that is, physicians, RNs, and SWPs, view HIS and CIS development participation. The responses were analyzed by managerial position, employment sector, and age group. Furthermore, we examined which types of professionals have participated in HIS and CIS development.

### RNs and SWPs Highly Aware of to Whom and How to Send Development-Related Feedback

The majority of RN (2204/3610, 61%) and SWP (556/990, 56%) respondents knew how and to whom to send development-related feedback; the respective proportion for physicians was 41% (1920/4683). The difference may be explained by mentoring or superuser and training programs during and after HIS implementations among RNs [9,53]. On the other hand, since the response rate among RNs and SWPs was relatively low, those who responded were probably more interested in ISs and thus more aware of HIS and CIS development than those who did not. The findings concur with our earlier studies, which have shown that physicians tend to be more critical towards their HIS compared with RNs and SWPs [2,5].

Leadership and its competence play an important role in the implementation of ISs and investment of resources in digitalization [22,54].

In this study, leaders in all professions and those with allotted working time for HIS or CIS development were more aware of feedback processes than others. These are usually responsible for the orientation of personnel, furthermore, they are often the ones to whom other personnel report development ideas or problems with HIS or CIS use. In all 3 professional groups the youngest were least aware of the feedback processes; whereas the youngest often have mentors who also help with HIS or CIS related problems, it is also probable that the currently available means are not suitable for the younger generations.

### Leaders Critical About Cooperation

Although leaders had more often dedicated working time and participated more often in the development of HISs or CISs than the others, they were critical about cooperation with vendors. Their information needs, informatics competencies, and partly also the ISs differ from those working in nonmanagerial positions [55]. The particularly negative viewpoints of physician leaders may be impacted by most of them regularly using HISs for direct patient care, unlike RN or SWP managers who mainly use HISs or CISs for managerial purposes [56]. The most recent study shows that RN managers are able to use HISs for their managerial duties. Due to poor system integration, they need to gather data from different systems for management, which wastes resources inefficiently [22].

### Those With Dedicated Working Time Less Dissatisfied With Vendor Cooperation

Those who had dedicated working time for HIS or CIS development were less dissatisfied with vendors’ interest and responsiveness to development ideas than those who had not participated at all. They are likely to be more aware of the development processes and timelines of their respective HISs or CISs. Furthermore, since they have been chosen by their respective organizations as participants in HIS or CIS development, their ideas are more likely to become realized. Earlier studies have also found that user participation increases acceptance and active use of HISs contributes to the acceptance and increased active use of HISs [57]. Although not all development ideas are suitable for execution and not all end users can be expected to spend considerable working time on HIS or CIS development, to achieve better engagement in the use of ISs, users need to experience that they are heard and understood [57].

### The Youngest Least Satisfied With Vendor Cooperation

Similar to the findings of our previous studies [1,4,6], the youngest were the most dissatisfied with vendors’ interest in feedback. This is a particularly important finding as it suggests that the current ways of engaging professionals will not become more suitable or even acceptable to future generations. Barchielli et al [58] also found that younger nurses rely on their colleagues’ opinions of health technology use, while older nurses rely on their own experiences.

### What Kinds of Users Have Participated in HIS or CIS Development?

Previous studies have shown that impactful participation in IS development requires dedicated working time [58]. Of those working in nonmanagerial positions, 72%‐82% responded that they have not participated at all in HIS or CIS development, whereas the respective proportion for leaders was 45%‐57%. Physician and RN leaders were most likely to have allotted working time for HIS development. Our findings agree with several studies suggesting that managerial viewpoints are likely to become overrepresented in IS development [59,60]. Although the data produced by the ISs is essential for leadership and management purposes, if the participating leaders are not engaged in clinical work or practice the solutions may end up not supporting the needs of frontline workers [61,62].

Health care professionals working in the private sector participated less than their public sector colleagues, among SWPs the differences were minimal. As the majority of Finnish private sector physicians work as private practitioners, their participation would usually result in decreased earnings. It is also likely that the lack of most complex patients in private healthcare reduces the need for HIS development.

In all 3 professional groups, the youngest participated the least. This may be because they are at the stage of learning the clinical content of their work and their employers may not want to invest their time in HIS or CIS development. Khairat et al [62] also report underrepresentation of physicians in specialization training in HIS development groups. Although not possessing advanced professional skills, the youngest are not burdened with old, often paper-based workflows, which could assist in redesigning processes and enhance the use of newer technologies [62].

### How to Improve Satisfaction in HIS and CIS Development

The development processes of large-scale complex systems such as HIS and CIS are typically dominated by cooperative activities involving multiple stakeholders [63-65]. Different information needs must be identified, prioritized, and communicated clearly enough to the IS designers and developers [65].

It remains challenging to increase user input and select appropriate participants and human-centered design methods through the different phases of the participatory development cycle [15,20]. Identifying other factors that influence user experiences, such as decisions made by regulators, policymakers, and administrators, may assist in developing better HISs and CISs [66]. Previous studies have recognized the importance of clinical informaticists who also use HISs or CISs in clinical work in communicating end users’ needs and feedback to designers and developers [6,67-75]. From the organizational perspective, the benefits can be seen beyond the IS implementation phase [76]: informatics competent social and health care professionals have been found to be able to improve patient safety and patient care outcomes [67]. In social welfare, however, this role is still being developed [74,75].

### Limitations

Our study has some limitations. First, compared with physicians, the lower response rates among RNs and SWPs are likely to have resulted in the selection of more involved and interested participants among these professional groups.

Second, the response rates were highest for physicians. The FMA, which was responsible for collecting the physician data, has a long history of conducting surveys among physicians, the results of which are used in FMA policies. However, the number of participating professionals from all 3 professional groups in the national level studies can be considered high compared to other similar studies [50].

The RNs questionnaire respondents, comprising nurses from various sectors including hospitals, health centers, private practice, and social care, were found to align with the target population according to Statistics Finland’s employment data for nurses, midwives, and community nurses [9].

During data collection with SWPs questionnaire, incomplete contact information in membership registries limited survey outreach. To compensate, survey invitations were distributed also through sector networks and social media. The study’s final sample comprised 990 SWP respondents, with an estimated response rate of 8%. It’s important to note this limitation when interpreting findings. Nonetheless, the sample size was considerable and exhibited diversity in age, service backgrounds, and geographical representation across Finland [51].

Third, our questionnaire did not cover how end users were involved in HIS or CIS development and whether the means of participation would impact satisfaction with the process and the end results. Further research is needed on best practices of user participation in the development of complex HIS and CIS systems.

### Conclusion

The fluent use of HISs and CISs is a prerequisite for efficient and safe health care and social welfare, as currently professionals spend a considerable part of their working time with ISs and rely on them as their primary source of information. User participation of all major professional groups—physicians, RNs and SWPs—and their involvement in development are essential for the success of complex HIS or CIS. Compared with RNs and SWPs, physicians appeared to be more critical towards IS vendors and the success of participatory HIS development. As even those with allotted working time were mostly dissatisfied with vendor cooperation, it is evident that simply allocating more end users’ working time for HIS and CIS development will not guarantee satisfaction; rather, dialogue between end users and developers needs improvement. New means are needed to better engage all end-user groups, particularly the youngest ones and those working in nonmanagerial positions.

## Supplementary material

10.2196/51495 Multimedia Appendix 1 Experiences of participation in system development.

## Abbreviations

CIS: client information system
FMA: Finnish Medical Association
HIS: health care information system
IS: information system
RN: registered nurse
SWP: social welfare professional
