# Supplementary material for: Comparisons of Physicians’, Nurses’, and Social Welfare Professionals’ Experiences With Participation in Information System Development: Cross-Sectional Survey Study
Source: JMIR Hum Factors. 2025 Jan 22;12:e51495. doi: 10.2196/51495 (PMC11780565; doi:10.2196/51495)
Supplement: Multimedia Appendix 1 [file humanfactors-v12-e51495-s001.docx]

# Table S1. Experiences of participation in system development

| **Participation experiences** | | **Knows how to send feedback** | **Vendor is interested** | **Desired manner** | **Quickly enough** |
| --- | --- | --- | --- | --- | --- |
|  |  | **Agree n (%)** | **Agree n (%)** | **Agree n (%)** | **Agree n (%)** |
| **Physicians** | leaders | 580 50,9% | 249 21,9% | 196 17,2% | 106 9,3% |
|  | others | 1340 37,8% | 527 14,9% | 431 12,2% | 301 8,5% |
| **RNs** | leaders | 316 **77,8%** | 144 35,5% | 87 21,4% | 49 12,1% |
|  | others | 1888 58,9% | 816 25,5% | 620 19,4% | 432 13,5% |
| **SWP** | leaders | 119 69,2% | 58 33,7% | 38 22,1% | 25 14,5% |
|  | others | 437 53,4% | 197 24,1% | 142 17,4% | 97 11,9% |
| **Physicians** | public | 1474 40,3% | 586 16,0% | 482 13,2% | 311 8,5% |
|  | private | 339 43,7% | 145 18,7% | 110 14,2% | 76 9,8% |
|  | other | 107 42,3% | 45 17,8% | 35 13,8% | 20 7,9% |
| RN**s** | public | 1877 61,0% | 815 26,5% | 585 19,0% | 407 13,2% |
|  | private | 278 61,0% | 123 27,0% | 105 23,0% | 64 14,0% |
|  | other | 49 62,8% | 22 28,2% | 17 21,8% | 10 12,8% |
| **SWPs** | public | 472 55,8% | 206 24,3% | 140 16,5% | 98 11,6% |
|  | private | 48 53,3% | 28 31,1% | 21 23,3% | 15 16,7% |
|  | other | 36 66,7% | 21 38,9% | 19 35,2% | 9 16,7% |
| **Physicians** | age group <35 | 312 32,9% | 119 12,5% | 100 10,5% | 79 8,3% |
|  | age group 35-44 | 473 38,9% | 187 15,4% | 165 13,6% | 109 9,0% |
|  | age group 45-64 | 510 43,9% | 222         19,1% | 166 14,3% | 101 8,7% |
|  | age group 55-64 | 609 46,3% | 238 18,1% | 192 14,6% | 114 8,7% |
| **RNs** | age group <35 | 380 51,4% | 169 22,9% | 137 18,5% | 104 14,1% |
|  | age group 35-44 | 533 64,0% | 225 27,0% | 164 19,7% | 104 12,5% |
|  | age group 45-64 | 721 65,1% | 309 27,9% | 212 19,1% | 142 12,8% |
|  | age group 55-64 | 563 61,1% | 253 27,5% | 192 20,8% | 129 14,0% |
| **SWPs** | age group <35 | 94 50,8% | 40 21,6% | 26 14,1% | 18 9,7% |
|  | age group 35-44 | 188 54,3% | 89 25,7% | 60 17,3% | 42 12,1% |
|  | age group 45-54 | 163 62,7% | 70 26,9% | 53 20,4% | 35 13,5% |
|  | age group 55-64 | 111 56,1% | 56 28,3% | 41 20,7% | 27 13,6% |
| **Physicians** | yes, with allotted working time | 137 68,5% | 62 31,0% | 43 21,5% | 20 10,0% |
|  | yes, no allotted working time | 488 53,2% | 213 23,2% | 168 18,3% | 97 10,6% |
|  | no | 1288 36,5% | 499 14,1% | 415 11,8% | 287 8,1% |
| **RNs** | yes, with allotted working time | **188 87,0%** | 114 52,8% | 80 37,0% | 44 20,4% |
|  | yes, no allotted working time | **449 77,4%** | 237 40,9% | 175 30,2% | 105 18,1% |
|  | no | 1567 55,7% | 609 21,6% | 452 16,1% | 332 11,8% |
| **SWPs** | yes, with allotted working time | **49 80,3%** | 27 44,3% | 22 36,1% | 15 24,6% |
|  | yes, no allotted working time | **189 71,9%** | 94 35,7% | 63 24,0% | 40 15,2% |
|  | no | 318 47,9% | 134 20,2% | 95 14,3% | 67 10,1% |
